# Changing the Smoking Trajectory: Evaluating the Impact of School-Based Tobacco Interventions on Changes to Susceptibility to Future Smoking

**DOI:** 10.3390/ijerph14101182

**Published:** 2017-10-05

**Authors:** Adam G. Cole, Wei Qian, Scott T. Leatherdale

**Affiliations:** School of Public Health and Health Systems, University of Waterloo, Waterloo, ON N2L 3G1, Canada; wei.qian@uwaterloo.ca (W.Q.); sleather@uwaterloo.ca (S.T.L.)

**Keywords:** adolescent, susceptibility, tobacco, evaluation studies, schools

## Abstract

School-based programs and policies can reduce student smoking rates. However, their impact on never-smoking students has not been investigated despite the clear transition between non-susceptible, susceptible, and ever tried smoking statuses. The objective of this paper was to examine the longitudinal student-level impact of six changes in school-based tobacco control programs and policies on student transitions in susceptibility to smoking over one year. Two multinomial logistic regression models identified the relative risk of a change in self-reported susceptibility to smoking or in trying a cigarette among never-smoking students in each of the six intervention schools compared to the relative risk among never-smoking students in control schools. Model 1 identified the relative risk of a change in smoking susceptibility status among baseline non-susceptible never smoking students, while Model 2 identified the relative risk of a change in smoking susceptibility status among baseline susceptible never smoking students. Students at some intervention schools were at increased risk of becoming susceptible to or trying a cigarette at one year follow-up. Intervention studies should examine changes to susceptibility to future smoking when evaluating impact to ensure that school-based tobacco control programs and policies do not negatively change the risk status of never-smoking students.

## 1. Introduction

Youth smoking rates within Canada have decreased substantially such that, by 2014/2015, only 9.7% of adolescents aged 15–17 years reported being current smokers; however, almost one-third of youth remained at risk of (i.e., susceptible to) future smoking [1]. There is a clear progression in stages of smoking from being a non-susceptible never smoker, to a susceptible never smoker, to trying a cigarette. Methods of measuring a never-smoking student’s likelihood of being a smoker in the future have been previously identified and validated through the construct know as smoking susceptibility [2,3]. As described by Pierce and colleagues [2,3], susceptibility to future smoking is determined by three questions used to identify a student’s intention to smoke cigarettes in the future; among never smoking students, susceptible never-smokers are at the highest risk of initiating cigarette smoking in the future [2,3].

The school environment has been widely accepted as an ideal setting in which to intervene with youth because a variety of students from different socio-economic backgrounds attend school for a large part of the day during the age range in which most people initiate cigarette smoking [4]. School-based interventions also affect all students, regardless of their smoking status (i.e., susceptible never smokers and current smokers). Identifying how school-based tobacco control interventions impact non-susceptible and susceptible never smoking students is important for understanding the effectiveness of these interventions at preventing smoking uptake and changing a student’s smoking trajectory. Various school-based tobacco control programs and policies have been implemented with varying degrees of success at reducing the smoking prevalence among students. A review of school-based tobacco prevention education programs found a significant reduction in smoking initiation [5]; however, evaluations of these programs do not identify the more nuanced changes to the risk status of non-smoking students, such as the transition from non-susceptible never smoker to susceptible never smoker which represents an increased risk of smoking in the future. To our knowledge, only a single study has examined the effect of prevention programs on student susceptibility to smoking and not only smoking initiation. The evaluation of this school-based tobacco prevention program in India found that students exposed to the prevention program were significantly less likely to be susceptible to future smoking [6,7].

In addition to tobacco prevention programs, schools may implement tobacco control policies that seek to restrict tobacco use on school property. Two reviews evaluated the impact of school-based tobacco control policies on reducing the prevalence of tobacco use among students [4,8]. Although cross-sectional studies indicate that schools with stronger tobacco control policies that prevent smoking on school property by staff [9] and schools that enforce smoking restrictions [10,11,12,13] have reduced odds of students smoking, the only longitudinal study to our knowledge that has been done has failed to support these cross-sectional findings [14]. In addition to limited longitudinal evidence, these studies have focused on individual smoking outcomes and have not examined the effect of implementing school-based tobacco control policies on non-smoking students’ susceptibility to future smoking. Given that school-based tobacco control policies are usually targeted to students that smoke, these policies may have a different impact on never-smoking students that are non-susceptible or susceptible to future smoking.

We previously reported the school-level results from 17 schools that changed their tobacco control policies or programs using two years of longitudinal school-level data and a difference-in-difference analytic approach [15]. These data identified six interventions that had a significant impact on the school-level prevalence of susceptible never-smokers. To our knowledge, there is currently a lack of experimental or quasi-experimental evidence worldwide that evaluates the effectiveness of school-based tobacco control programs and policies [8], especially among susceptible and non-susceptible never smokers. As a follow-up to our previous school-level analysis, the objective of this paper is to examine the longitudinal student-level impact of these six interventions on four possible student transitions in susceptibility to smoking over one year, controlling for relevant student- and school-level correlates.

## 2. Materials and Methods

This study evaluated the real-world impact of six school-based tobacco control interventions in Ontario, Canada using data from the COMPASS study. COMPASS is a school-based cohort study designed to evaluate the influence of changes to programs, policies, and the school environment on various health behaviours [16]. A full description of the study methods is available in print [16] or online (www.compass.uwaterloo.ca). Briefly, a convenience sample of 43 secondary schools in Ontario, Canada that approved of active information-passive consent parental permission protocols were recruited to participate in COMPASS. The COMPASS study received ethics approval from the University of Waterloo Research Ethics Board, as well as participating school board review panels.

The current analysis uses data from a cohort of never-smoking students in grades 9 to 11 that attended 32 secondary schools in Ontario, Canada (*n* = 6 intervention schools and *n* = 26 control schools; 11 schools were excluded as described below). All students enrolled in the participating schools at baseline (Year 1, 2012/2013, *N* = 30,147) were eligible and invited to participate in the survey. The response rate at baseline was 80.2%. Similarly, at one year follow-up (Year 2, 2013/2014) all students in the participating schools were eligible and invited to participate in the survey (*N* = 29,945). The response rate at follow-up was 78.2%. Missing respondents in both years resulted primarily from scheduled spares or absenteeism at the time of the data collection; few respondents were missing due to student or parent refusal (~1% in each year). As described elsewhere [17,18], the longitudinal sample of students in grades 9 to 11 (*N* = 11,049) was isolated using a unique identifier that was linked between baseline and follow-up and was generated following student responses to six questions.

For this analysis, changes to self-reported susceptibility to smoking were identified among never-smoking students between baseline and follow-up. Students that reported having ever tried a cigarette at baseline (*n* = 2078) and with missing outcomes at baseline (*n* = 75) or follow-up (*n* = 62) were excluded from this analysis, leaving a final sample of 8,834 never smoking students at baseline.

### 2.1. Measures

#### 2.1.1. Student-Level Measures

Self-reported smoking susceptibility and current smoking measures were collected using the COMPASS questionnaire (Cq), a paper-based survey that collects student-level data pertaining to multiple health behaviours (e.g., tobacco use, physical activity, diet, substance use, etc.), correlates of the behaviours, and demographic characteristics. Data were collected during a single class period at each school. Consistent with previously validated measures [2,3], smoking susceptibility among never smokers at baseline and follow-up was assessed on the Cq using three questions: “Do you think in the future you might try smoking cigarettes?”; “If one of your best friends were to offer you a cigarette, would you smoke it?”; and, “At any time during the next year do you think you will smoke a cigarette?”. Consistent with previous research [2,3], students that responded “definitely not” to all three questions were identified as non-susceptible never smokers, while students that provided any other combination of responses were identified as susceptible never smokers. Ever smoking at follow-up was measured by asking students “Have you ever tried cigarette smoking, even just a puff?” Students that reported ever trying a cigarette were classified as ever smokers.

#### 2.1.2. School-Level Measures

Changes to school-level programs and policies related to tobacco control between baseline and follow-up were measured using the COMPASS School Programs and Policies questionnaire (SPP). The SPP is a paper-based survey that measures the presence or absence of policies, practices, and resources relevant to multiple health behaviours (e.g., tobacco use, physical activity, diet, substance use, etc.), and annual changes to these school policies, practices, or resources. It is important to note that changes to programs, policies, or resources were initiated by school stakeholders after receiving data about the health behaviours of students at their schools; changes to programs and policies relevant to tobacco control could have been made in response to high smoking rates at the school and may not have been informed by best practices. The SPP was completed by the school administrator(s) most knowledgeable about the school program and policy environment and was collected at the time of the school’s student-level data collection.

Consistent with our previous study [15], we used data from the SPP administered at follow-up to identify any changes to programs and policies relevant to tobacco control between baseline and follow-up. At follow-up, the SPP provided a summary of the relevant tobacco control programs and policies identified during the previous year at each school and asked the administrators to report whether the policies, practices, environment, and relationships from baseline were still in place at follow-up and whether any new policies, practices, environment changes, or relationships were implemented between baseline and follow-up. It was left up to the school administrator to include as much information about the change in policy, practice, or environment as possible. When necessary and possible, COMPASS staff also followed-up with each school to verify the information provided in the SPP and clarify any changes to school policies, practices, or resources.

### 2.2. Description of Interventions

We previously identified six tobacco program or policy interventions that had a significant impact on the school-level prevalence of susceptible never smokers [15]. A brief description of these interventions (based on information provided by the school administrator in the SPP) is provided below.

School 6 (Effective and enforced tobacco control policy): School administration began to consistently enforce the ban on smoking on school property. Students caught smoking on school property were fined for a first offence and suspended from school for a second offence. No information was provided on the fine amount or the number of students that were caught and penalized.

School 7 (Effective and enforced tobacco control policy): A Tobacco Enforcement Officer was called to enforce the ban on smoking on school property. Punishment for offences was at the discretion of the Tobacco Enforcement Officer. No information was provided on the types of penalties delivered or the number of students that were caught and penalized.

School 8 (Effective and enforced tobacco control policy): The Public Health Unit was responsible for sending letters home to parents of students caught smoking on school property. Students were also fined $300–400. No information was provided on the number of students that were caught and penalized.

School 12 (Cessation intervention): Two teachers within the school created their own smoking cessation program. No additional details about the program were provided.

School 15 (Staff training): Teachers were encouraged to receive professional development training related to tobacco prevention (including attending conferences, workshops or presentations). No information was provided on the types of training or number of teachers that attended.

School 16 (Staff training): Teachers were no longer allowed to receive professional development training related to tobacco prevention using school resources. No information was provided on the types of training or number of teachers that attended previously.

### 2.3. Analysis

Based on data from the SPP, schools that reported a change in tobacco control programs and/or policies between baseline and follow-up were identified as intervention schools, while those that did not report a change in tobacco control programs and/or policies between baseline and follow-up were identified as control schools and were grouped together. The current analyses focused on identifying the relative risk of a change in self-reported susceptibility to smoking or in trying a cigarette among never-smoking students between baseline and follow-up in each of the six intervention schools compared to the relative risk of a change to self-reported susceptibility to smoking or in trying a cigarette among never-smoking students in control schools (*n* = 26); 11 schools made a change to their tobacco control program or policy environment between baseline and follow-up that did not result in a significant school-level change in the prevalence of susceptible never smokers and were excluded from these analyses.

Our previous findings identified significant increases in the school-level prevalence of susceptible never smokers at Schools 6–8 and 15, and significant decreases in the school-level prevalence of susceptible never-smokers at Schools 12 and 16. These changes could be a result of non-susceptible never smokers becoming susceptible to future smoking (resulting in an increased prevalence of susceptible never smokers) or susceptible never smokers trying smoking (resulting in a decreased prevalence of susceptible never smokers). We used logistic regression to clarify the student-level transitions in susceptibility to future smoking occurring in these schools. We initially created four multi-level binary logistic regression models using GEE in SAS (SAS Institute Inc., Cary, NC, USA). Through these models, we observed that the correlation within schools was not significant. Therefore, we simplified the modelling technique and created two multinomial logistic regression models.

The two multinomial logistic regression models identified the impact of six interventions on susceptibility to smoking at follow-up, modelling the relative risk of changing smoking susceptibility status between baseline and follow-up versus no change in smoking susceptibility status. Model 1 identified the relative risk of a change in status among baseline non-susceptible never smoking students and Model 2 identified the relative risk of a change in status among baseline susceptible never smoking students. The outcome for both models was the response to susceptibility to smoking at follow-up with three possible responses: non-susceptible never smoker, susceptible never smoker, and ever smoker. While non-susceptible never smokers were the reference group for Model 1, susceptible never smokers were the reference group for Model 2. Six schools with different interventions were included as six treatment groups in each of the two models; schools that did not report a change in tobacco control programs and/or policies were identified as control schools and were grouped together. Both models controlled for age at baseline, gender, race/ethnicity, the reported difference in the number of close friends that smoked cigarettes between baseline and follow-up, and school location (urban/rural). All analyses were conducted in SAS 9.4 (SAS Institute Inc., Cary, NC, USA).

## 3. Results

Baseline characteristics of students in control and intervention schools are presented in Supplementary Table S1. The prevalence of susceptible never smoking students was not significantly different between control and intervention schools. Baseline school-level characteristics of control and intervention schools are presented in Supplementary Table S2. The distribution of characteristics of control and intervention schools are relatively similar.

Table 1 presents the changes in susceptibility in the longitudinal sample between baseline and follow-up among baseline never smokers for control and intervention schools. There was some variation in the prevalence of susceptible never smokers at control and intervention schools at baseline and considerable variation in the prevalence of susceptible never smokers and ever smokers at control and intervention schools at follow-up.

Table 2 presents the multinomial regression analyses evaluating the relative risk ratio of a non-susceptible never smoking student becoming susceptible to smoking and starting smoking at follow-up for each intervention school, controlling for relevant covariates. The intervention in School 8 (effective and enforced tobacco control policy) had an undesirable effect compared to control schools and significantly increased the risk that a non-susceptible never smoker became a susceptible never smoker and tried smoking at follow-up rather than remaining non-susceptible to future smoking. Similarly, the intervention in School 7 (effective and enforced tobacco control policy) had an undesirable effect compared to control schools and significantly increased the risk that a non-susceptible never smoker tried smoking at follow-up rather than remaining non-susceptible to future smoking.

Table 3 presents the multinomial regression analyses evaluating the relative risk ratio of a susceptible never-smoking student becoming non-susceptible to smoking and starting smoking at follow-up for each intervention school, controlling for relevant covariates. The intervention in School 6 (effective and enforced tobacco control policy) had a desirable effect compared to control schools and significantly reduced the risk that a susceptible never smoker ever tried smoking at follow-up rather than remaining susceptible to future smoking. The intervention in School 12 (cessation intervention) had an undesirable effect compared to control schools and significantly increased the risk that a susceptible never smoker ever tried smoking at follow-up rather than remaining susceptible to future smoking.

## 4. Discussion

The results from this quasi-experimental study indicate that some interventions had a positive impact on student risk of smoking over time, while other interventions had a negative impact on student risk of smoking over time. Our results indicate that the interventions at School 7 and School 8 had a negative impact on student risk of smoking and increased the risk that non-susceptible students progressed to become susceptible to future smoking and try smoking. The intervention at School 7 involved enforcement by the Tobacco Enforcement Officer, while the intervention at School 8 involved sending a letter home to parents of students that were caught smoking on school property. Students were also fined when caught smoking on school property. Although previous cross-sectional evidence suggests that consistent policy enforcement is important for reducing the number of students that smoke at school [10,11,12,13], and our previous school-level analysis indicated that these types of interventions reduced the prevalence of current smokers at schools [15], the longitudinal student-level data suggest that there may be unintended consequences for never-smokers. According to reactance theory, restricting individual freedoms, such as by implementing school smoking bans, may reduce the effectiveness of the health promotion message and lead to unintended consequences, such as increased smoking [19,20]. Although it was beyond the scope of the SPP to collect process evaluation data, it is also possible that the Tobacco Enforcement Officer did not consistently enforce the policy, parents never saw the letters that were sent home, or students did not end up paying the fine. These factors may have reduced the effectiveness of this intervention, increasing the risk of future smoking for non-smoking students.

In contrast, the intervention at School 6 had a positive impact on student risk of smoking and reduced the risk that susceptible students tried smoking at follow-up. The intervention at School 6 involved consistent enforcement of the school tobacco policy by school administration. Although it cannot be confirmed from the SPP data, it is possible that the need for this intervention was identified by school administration. This may have contributed to its positive impact since school administration would be more mindful of consistently enforcing the school tobacco policy given their role and concern for student health. Additional evidence is needed to compare the relative effectiveness of external versus internal individuals responsible for enforcement activities.

The cessation intervention at School 12 had a negative impact on never-smoking students. These analyses indicate that susceptible never smokers were at a significantly higher risk of ever trying smoking at follow-up. These data illustrate the importance of examining the impact of school tobacco control interventions on student susceptibility to smoking as some interventions may have unintended consequences. It is unclear why susceptible never smokers were at a higher risk to try smoking in this teacher-led intervention. Further evidence is needed to identify the impact of cessation interventions on students that do not smoke but are at higher risk of experimentation.

Overall, these results emphasize the need for knowledge translation and exchange tools between researchers and school stakeholders. Many school administrators do not have training in health program planning, policy implementation, or evaluation, and health promotion activities may be outside the scope of the school mandate. None of these interventions took a comprehensive approach to addressing tobacco use, but rather used single, one-off tactics that may be counter to best practice. For example, punitive approaches (such as fines or suspending students caught smoking on school property) may actually increase deviant behaviours (such as cigarette smoking) among certain groups [21]. Additional guidance is necessary to help school administrators make evidence-based, informed decisions about programs and policies to improve student health.

The quasi-experimental design of this study provides evidence for the real-world impact of each of these interventions, and the longitudinal data from this cohort study illustrate how these interventions impact student-level behaviours over time. Furthermore, this study fills an important knowledge gap with respect to how changes in school-level tobacco control programs and policies affect the risk of future smoking among never-smoking students. Previous research has focused on the effect of these programs and policies on smoking outcomes such as reducing the likelihood of smoking initiation or progression [4,8] and has neglected susceptibility to future smoking as an outcome. Similar to other school-based studies, we were limited to using self-reported measures of smoking behaviour; however, both the measures of smoking susceptibility [22,23,24] and ever smoking [25] were consistent with previous measures, allowing for comparison with other studies. Student drop-out is a common concern in longitudinal studies; however, given that students that use cigarettes and other substances are more likely to drop out of longitudinal studies [18,26], the current results may underestimate the impact of school-level interventions. We were also limited in the process and implementation data that were available for each intervention school and in most cases a complete description of the intervention was unavailable. Finally, given the quasi-experimental design of this study, we were unable to include a clean control group or control for external factors that could impact the fidelity of the interventions. Environmental factors within the school environment, such as demographic characteristics of students, school-level socioeconomic status, and tobacco retailer density could also impact the effectiveness of interventions. Although we attempted to control for common factors that are known to influence student smoking, including student-level gender and race, the change in the number of friends that smoke, and school location (urban/rural), many other factors may have influenced intervention fidelity and success. Given that the distribution of characteristics of control and intervention schools were relatively similar (as shown in Supplementary Table S2), it is likely that external school-level characteristics had a minimal impact on the results. Future studies could attempt to replicate these findings using a randomized design.

## 5. Conclusions

These data illustrate the importance of examining changes to susceptibility to future smoking when evaluating school-level tobacco control programs and policies, and the value of longitudinal data. Although many students progressed towards a higher risk of smoking and some tried smoking cigarettes, few schools changed the tobacco control program or policy environment over one year. Few interventions in this study had a significant positive influence on student susceptibility to smoking. Future evaluations should ensure that school-based tobacco control programs and policies do not negatively change the risk status of never-smoking students. Additional evidence is necessary to determine the components that successfully reduced the risk of future smoking among never-smoking students.

## Figures and Tables

**Table 1:** Changes in smoking susceptibility in the longitudinal sample between baseline and follow-up among baseline never smokers according to intervention group, 2012–2014 COMPASS study, Ontario, ON, Canada.

Intervention Group	Status at Baseline		Status at Follow-up		
	Non-Susceptible Never Smoker (%)	Susceptible Never Smoker (%)	Non-Susceptible Never Smoker (%)	Susceptible Never Smoker (%)	Ever Smoker (%)
Control	73.0	27.0	65.7	21.9	12.3
Intervention (overall)	71.6	28.4	64.3	23.7	12.1
School 6	69.9	30.1	57.5	35.6	6.8
School 7	74.2	25.8	61.5	24.2	14.3
School 8	66.4	33.6	49.0	31.0	20.0
School 12	71.8	28.2	70.8	18.1	11.1
School 15	74.4	25.6	68.9	22.0	9.1
School 16	68.3	31.7	63.4	23.9	12.7

**Table 2:** Multinomial logistic regression analyses (Model 1) evaluating the impact of six school-specific tobacco interventions implemented between baseline and follow-up among baseline non-susceptible never smokers, 2012–2014 COMPASS study, Ontario, ON, Canada.

Parameter	Relative Risk Ratio of Becoming Susceptible vs. Remaining Non-Susceptible to Smoking (95% CI)	Relative Risk Ratio of Ever Smoking vs. Remaining Non-Susceptible to Smoking (95% CI)
Effective and enforced tobacco control policies		
School 6	1.50 (0.70, 3.22)	0.71 (0.21, 2.42)
School 7	1.38 (0.85, 2.23)	1.88 (1.05, 3.38) **
School 8	2.11 (1.21, 3.68) ***	2.19 (1.13, 4.26) **
Cessation intervention		
School 12	0.81 (0.51, 1.29)	0.48 (0.22, 1.06) *
Staff training		
School 15	1.18 (0.82, 1.68)	0.73 (0.40, 1.32)
School 16	0.90 (0.51, 1.58)	1.06 (0.54, 2.06)

Analysis models the relative risk that a non-susceptible never smoker became a susceptible never smoker or started smoking versus remained a non-susceptible never smoker in each intervention school (*n* = 6) relative to control schools (*n* = 26), controlling for age at baseline, gender, race, difference in number of friends smoking between baseline and follow-up, and school location (urban/rural). * *p* < 0.1; ** *p* < 0.05; *** *p* < 0.01.

**Table 3:** Multinomial logistic regression analyses (Model 2) evaluating the impact of six school-specific tobacco interventions implemented between baseline and follow-up among baseline susceptible never smokers, 2012–2014 COMPASS study, Ontario, ON, Canada.

Parameter	Relative Risk Ratio of Becoming Non-Susceptible vs. Remaining Susceptible to Smoking (95% CI)	Relative Risk Ratio of Ever Smoking vs. Remaining Susceptible to Smoking (95% CI)
Effective and enforced tobacco control policies		
School 6	0.29 (0.08, 1.04) *	0.18 (0.05, 0.83) **
School 7	0.99 (0.49, 1.99)	1.25 (0.58, 2.69)
School 8	0.73 (0.32, 1.64)	0.90 (0.42, 1.94)
Cessation intervention		
School 12	1.50 (0.85, 2.63)	2.11 (1.15, 3.86) **
Staff training		
School 15	1.42 (0.87, 2.31)	1.07 (0.59, 1.93)
School 16	0.87 (0.46, 1.64)	0.59 (0.29, 1.18)

Analysis models the relative risk ratio that a susceptible never smoker became a non-susceptible never smoker or ever tried smoking versus remained a susceptible never smoker in each intervention school (*n* = 6) relative to control schools (*n* = 26), controlling for age at baseline, gender, race, difference in number of friends smoking between baseline and follow-up, and school location (urban/rural). * *p* < 0.1; ** *p* < 0.05.

## Supplementary Materials

The following are available online at www.mdpi.com/1660-4601/14/10/1182/s1, Table S1: Baseline characteristics of students in control and intervention schools, 2012–13 COMPASS study, Ontario, ON, Canada, Table S2: Baseline school-level characteristics of control and intervention schools, 2012–13 COMPASS study, Ontario, ON, Canada.
