# Supplementary material for: Changing the Smoking Trajectory: Evaluating the Impact of School-Based Tobacco Interventions on Changes to Susceptibility to Future Smoking
Source: Int J Environ Res Public Health. 2017 Oct 5;14(10):1182. doi: 10.3390/ijerph14101182 (PMC5664683; doi:10.3390/ijerph14101182)
Supplement: Supplementary file 1 [file ijerph-14-01182-s001.pdf]

Table S1. Baseline characteristics of students in control and intervention schools, 2012–13 COMPASS study, Ontario, ON, Canada.

Characteristic at Baseline		Control Schools	Intervention Schools (Overall)	*p*-Value
Susceptibility	Non-susceptible	73.0	71.6	0.295
	Susceptible	27.0	28.4	
Age	13 years	1.3	0.4	0.027
	14 years	30.3	31.0	
	15 years	35.4	36.2	
	16 years	25.9	24.8	
	17 years	6.4	7.4	
	18 years	0.7	0.2	
Gender	Female	54.4	51.9	0.113
	Male	45.6	48.1	
Ethnicity	White	68.2	70.8	<0.001
	Black	3.5	1.6	
	Asian	6.1	4.2	
	Off-Reserve Aboriginal	0.8	2.3	
	Latin American/Hispanic	1.3	2.5	
	Other/ Missing	20.2	18.6	
Number of friends that smoke	None	79.3	74.5	0.003
	1 friend	11.1	14.1	
	2 friends	5.6	6.3	
	3 friends	2.0	2.2	
	4 friends	0.4	0.9	
	5 friends or more	1.5	2.0	

Table S2. Baseline school-level characteristics of control and intervention schools, 2012–13 COMPASS study, Ontario, ON, Canada.

Characteristic at Baseline		Control Schools	Intervention Schools (Overall)
School-level prevalence	Non-susceptible never smokers	59.7	55.1
	Susceptible never smokers	22.1	21.9
	Current smokers	2.4	3.4
School-level socioeconomic status (SES)	$25,000–50,000	15.4	16.7
	$50,001–75,000	57.7	66.7
	$75,001–100,000	19.2	16.7
	>$100,000	7.7	0.0
School location	Large urban area	53.8	50.0
	Medium urban area	11.5	0.0
	Small urban area/ rural area	34.6	50.0
